# Compound adsorption device in pathogen reduction technology removes multiple drugs from plasma components

**DOI:** 10.1111/trf.18485

**Published:** 2025-11-26

**Authors:** Briana Greenwall, Kathryn Reeder, Waseem Qais Anani

**Affiliations:** ^1^ ARUP Laboratories Salt Lake City Utah USA; ^2^ University of Utah Salt Lake City Utah USA

**Keywords:** CAD, compound adsorption device, donor medications, pathogen reduction, plasma donation, PRT

## Abstract

**Background:**

Blood donor eligibility policies may exclude individuals based on medication use, but the donor health questionnaire may not reliably capture all prescriptions. This study evaluates the capacity of the compound adsorption device (CAD) within the INTERCEPT Blood System Pathogen Reduction Technology (PRT) for plasma to remove exogenous testosterone and high doses of two drugs on the FDA donor deferral list.

**Study Design and Methods:**

Plasma was manufactured from male whole blood donors undergoing testosterone replacement therapy (TRT) or whole blood donors with no medication history to be supplemented with supratherapeutic concentrations of apixaban, a common anticoagulant, and escalating doses of emtricitabine and tenofovir (FTC&TFV) to note any saturating effects of removal. Plasma units were treated with INTERCEPT PRT only using the amotosalen and CAD steps without photochemical activation. Quantitative assays analyzed pre‐ and post‐CAD samples. Statistical analysis was performed using a Wilcoxon signed‐rank test and one‐way ANOVA.

**Results:**

The CAD reduced the mean free and total testosterone by 75% (pre‐CAD 107.4, post‐CAD 26.8 pg/mL) and 74% (pre‐CAD 352.2, post‐CAD 85.7 ng/mL), respectively. Apixaban was removed below the limit of detection (pre‐CAD mean 420, post‐CAD mean <23 ng/mL). Regardless of the initial dose, FTC&TFV concentrations decreased by 100% and 98%, respectively.

**Conclusion:**

The INTERCEPT PRT plasma kit CAD effectively removed multiple drug classes, suggesting a potential role in mitigating drug contamination in transfusable plasma products. These findings may support the reconsideration of some medication‐based donor deferrals where the risk–benefit of donor safety and product efficacy favors collection.

**Abbreviations**
CAD: compound adsorption device
FFP: fresh frozen plasma
FTC&TFV: emtricitabine and tenofovir
PrEP: pre‐exposure prophylaxis
PRT: pathogen reduction technology
TRT: testosterone replacement therapy

## 1 INTRODUCTION

A significant proportion of the general US population takes prescription drugs, which may affect donor eligibility.^1^ A Canadian study reviewing ~843,000 screened donors found that one‐third took at least one medication.^2^ As blood centers encounter these individuals, deferrals may solely be based on the medications being taken. For those drugs that are permissible, there is an unknown impact on transfusion recipients.

Health regulators set policies to decrease transfusion recipient secondhand exposure to donor medications.^3^, ^4^ The US Food and Drug Administration (FDA) prohibits blood donation for some medications that may broadly affect the efficacy of a collected product or are teratogenic. However, blood collectors determine the eligibility for a vast majority of medications not outlined by the FDA. This leads to policy variations within the United States and internationally.^3^, ^4^ Some have advocated for deferral policies factoring in the drug half‐life and maximal plasma concentration to prevent unnecessary deferrals.^5^, ^6^

Pathogen reduction technology (PRT) reduces transfusion‐transmitted infectious disease risks, but little has been done to address medication‐related contamination of blood products beyond deferral policies.^7^ The United States has one PRT system FDA‐approved for use with platelets and/or plasma: amotosalen/UVA (INTERCEPT Blood System, Cerus Corporation, Concord, CA). We previously showed that supraphysiologic free and total testosterone levels in donor plasma were removed by the compound adsorption device (CAD), designed to remove excess amotosalen and photoproducts, in the INTERCEPT kits for platelets.^8^, ^9^ From these data, we hypothesized that the INTERCEPT plasma kits, employing a different CAD configuration, may also absorb medications onto the polystyrene matrix through predominantly non‐covalent bonds.^10^ We evaluated the removal of exogenous testosterone; high doses of apixaban—a commonly used oral anticoagulant; and escalating doses of emtricitabine and tenofovir (FTC&TFV)—to demonstrate an effect on dual acting drugs and a newer medication to the FDA deferral list.^4^, ^11^

## 2 STUDY DESIGN AND METHODS

The University of Utah Institutional Review Board approved the study to collect donor data, treat donor fresh frozen plasma with INTERCEPT PRT, and perform testing. Eligible male donors over 18 years taking testosterone replacement therapy (TRT) were selected for free and total testosterone measurements. Plasma units from donors without a history of taking apixaban or emtricitabine and tenofovir (FTC&TFV) were selected. Information about donor age, gender, donor health questionnaire answers, and medication list was collected for the study.

Fresh frozen plasma (FFP) was manufactured from 450 mL of leukocyte‐reduced CPDA‐1 whole blood. Plasma units were frozen at −80°C until thawing for drug spiking (apixaban and FTC&TFV) and drug removal by the CAD. ABO identical FFP was thawed and pooled to meet the minimum volume requirements for pathogen reduction. Each FFP unit was sterile docked to the line with the amotosalen pouch until the pool was approximately 600 mL in the illumination container. To prevent any confounding effects from the illumination step, the amotosalen was added to the plasma and passed through the CAD into the collection container—the plasma was not illuminated. Donor FFP was spiked with apixaban (Sigma‐Aldrich, St. Louis, MO; Catalog number SML3285) above the *C*_max_ (peak serum concentration of the drug) and close to the upper limit of testing (430 ng/mL).^12^ Emtricitabine (Sigma‐Aldrich, St. Louis, MO; Catalog number PHR2120) and tenofovir (Sigma‐Aldrich, St. Louis, MO; Catalog number PHR1592) were added at increasing concentrations well over the *C*_max_ to evaluate the capacity of the CAD to remove the drugs.^13^ Ten pools were made from TRT donors and five pools from donors not reporting medications were manufactured for apixaban and FTC&TFV spiking each. Two samples were collected from each pool: when pooled in the illumination container (pre‐CAD) and following gravity‐dependent flow through the CAD (post‐CAD).

Aliquots were taken for pre‐CAD and post‐CAD drug measurements and frozen at −80°C until testing. Free and total testosterone were measured using equilibrium dialysis high‐performance liquid chromatography tandem mass spectrometry at ARUP Laboratories (Salt Lake City, UT). Apixaban concentrations were measured with a chromogenic assay at ARUP Laboratories with a testing range of 23–430 ng/mL. The FTC&TFV were extracted from plasma and quantitatively assessed by liquid chromatography tandem mass spectrometry with a lower limit of detection for FTC at 12,500 ng/mL and TFV at 5 ng/mL at the University of North Carolina at Chapel Hill Center for AIDS Research Clinical Pharmacology and Analytical Chemistry Core (Chapel Hill, NC). Pre and post samples were compared by non‐parametric one‐tailed *t*‐test (Wilcoxon signed‐rank test). Non‐parametric testing does not assume a Gaussian distribution of data, and it is less likely to be affected by large variations in small sample sizes and less influenced by outliers. Medications were added to plasma and thus had a skewed distribution. Free and total testosterone were analyzed by one‐way ANOVA (Friedman test). All analyses were completed with GraphPad Prism (version 10.2.1, GraphPad Software, Inc., La Jolla, CA).

## 3 RESULTS

### 3.1 Efficacy of drug removal by pathogen reduction system

Ten pools from TRT donors were significantly reduced by 75% for free and 74% for total testosterone post‐CAD (Table 1). There was a significant mean reduction in apixaban by 95%, emtricitabine by 100%, and tenofovir by 98% (Table 2). All samples were below the level of detection for apixaban (23 ng/mL). Four of the five samples for emtricitabine were below the limit of quantification (5 ng/mL). Five pools of increasing concentrations of emtricitabine and tenofovir well above the *C*_max_ for each drug had similar post‐CAD levels (Table 3). The lower limits were used in the statistical calculations when falling below the limit of detection/quantification.

**TABLE 1:** Free and total testosterone pre‐ and post‐CAD.

Testosterone	Pre‐CAD; mean ± SD (range); *n* = 10	Post‐CAD; mean ± SD (range); *n* = 10	Percent change	*p*	Reference range
Free (pg/mL)	107.4 ± 21.43 (75.70–142.60)	26.8 ± 7.99 (13.60–36.00)	−75%	.0065	47–244
Total (ng/mL)	325.2 ± 27.4 (181.1–415.8)	85.7 ± 10.6 (33.1–122.6)	−74%	.0065	300–1080

**TABLE 2:** Summary of apixaban, emtricitabine, and tenofovir concentrations pre‐ and post‐CAD.

	Pre‐CAD; mean ± SD (range)	Post‐CAD; mean ± SD (range)	Mean percent change	*p*	*C*_max_
Apixaban (ng/mL) *n* = 5	420 ± 13 (400–430)	<23 ± 0^a^	−95%	.0312	104.7
Emtricitabine (ng/mL) *n* = 5	852,400 ± 423,957 (286,000–1,440,000)	11.40 ± 14.31^b^ (5–37.00)	−100%	.0312	1800 ± 720
Tenofovir (ng/mL) *n* = 5	135,840 ± 69,265 (45,200–233,000)	1766 ± 134.50 (1630–1990)	−98%	.0312	300 ± 90

^a^ All samples were below the limit of detection (23 ng/mL).

^b^ Four of five samples were below the limit of quantification (5 ng/mL).

**TABLE 3:** Emtricitabine and tenofovir escalating concentrations pre‐ and post‐CAD.

	Pre‐CAD	Post‐CAD	Percent change
Emtricitabine concentration (ng/mL)			
Pool 1	286,000	BLQ^a^	100%
Pool 2	699,000	BLQ^a^	100%
Pool 3	817,000	BLQ^a^	100%
Pool 4	1,020,000	37	100%
Pool 5	1,440,000	BLQ^a^	100%
Tenofovir concentration (ng/mL)			
Pool 1	45,200	1990.00	−96%
Pool 2	105,000	1630.00	−99%
Pool 3	136,000	1750.00	−99%
Pool 4	160,000	1750.00	−98%
Pool 5	233,000	1710.00	−99%

^a^ Below the limit of quantification: the lowest limit of quantification was 5 ng/mL.

## 4 DISCUSSION

We previously found that the CAD within the INTERCEPT PRT for platelets was effective in removing testosterone from plasma.^8^ This study also showed testosterone was effectively removed and extracted apixaban and FTC&TFV using the INTERCEPT PRT for plasma kits. The polystyrene matrix in the CAD, regardless of the configuration, removed multiple drug classes even with amotosalen present.^9^ This may help mitigate medication‐related donor deferrals, which rose from 2.9% in 2019 to 4.5% in 2021 in the United States.^14^ This process may also safeguard against the 11% of donors on undisclosed medications later identified by serum toxicology analysis.^15^

Comparing the testosterone results across the studies provides insight into drug removal rates. CAD incubation times differed significantly: platelet components incubate for >4 h with a wafer CAD device, while the plasma components flow through a disc‐shaped CAD by gravity. Based on the rate of plasma flow we observed, the plasma CAD is in contact with the medications for less than a minute before passing into the plasma storage container.^16^, ^17^ Platelet PRT kits yielded mean reductions of 84% and 88% for free and total testosterone (66.4 pg/mL, 187.0 ng/mL) compared with 75% and 74% (26.8 pg/mL and 85.7 ng/mL) with the plasma PRT kits. Although the percentage change is smaller, the absolute values are lower in this study. The prior study screened and selected the highest testosterone values, while the current study chose TRT donors at random, resulting in lower mean starting concentrations. Differences may reflect variability in extraction efficiency, contact time, and nonlinear drug removal kinetics.

Apixaban, a commonly prescribed oral anticoagulant, was selected because it is subject to an FDA‐mandated deferral to prevent donor bleeding and preserve plasma efficacy.^4^, ^11^, ^18^ Apixaban is an ideal pilot anticoagulant, because it reversibly binds to Factor Xa compared with drugs that irreversibly bind or alter platelets (e.g., aspirin) and/or plasma (e.g., vitamin K antagonists). Spiking plasma with apixaban well above therapeutic levels resulted in post‐CAD values below the detection limit. Pathogen reduction using a CAD preserves the efficacy of the product but does not improve the bleeding risk to the donor. A retrospective study reviewed donors on anticoagulants with post‐donation hematomas measuring at least 2 inches in diameter, finding that anticoagulation increased the observed rate from 0.06% to 0.52% (*p* < 0.0001).^19^ As the donor base continues to decline, the risk–benefit of collecting donors on anticoagulants may favor removing the mandated deferral in combination with INTERCEPT PRT.

We also tested a recent medication on the deferral list at extraordinarily high concentrations to observe any saturation effects in the CAD. Pre‐exposure prophylaxis (PrEP) is an antiretroviral dual‐medication used by HIV‐negative individuals to reduce the risk of contracting HIV after exposure.^13^ FTC&TFV is an oral preparation that may cause transient or delayed infections below the limit of detection with donor screening assays, but the medication poses no risk to transfusion recipients.^20^ The two‐drug combination was an optimal test case, since there is a robust quantification assay, even at high concentrations.^21^ The mean dose of emtricitabine pre‐CAD was over 473,000 times over the *C*_max_ and tenofovir was over 452,000 times over the *C*_max_.^13^ Surprisingly, no matter the concentration of each drug, the final concentration was similar, suggesting there was an ample capacity for removal (i.e., within the region of independence).^10^ It is important to note that all the post‐CAD tenofovir samples were above the *C*_max_, but we could not extrapolate if a lower starting concentration would cause an undetectable drug level or if this related to the drug affinity for the CAD material. Polystyrene formulations with high surface area to volume and kinetics favoring hydrophobic binding may explain the almost complete removal of the drugs^10^; however, future studies should explore removal with physiological doses and the possible variability in drug removal using a CAD.

Notably, this study did not illuminate the plasma component so that photochemical degradation was not a confounding factor. However, both drugs are noted to resist photochemical degradation.^22^, ^23^ Amotosalen was included to evaluate potential competitive binding, but we did not measure amotosalen directly. The lack of FTC&TFV removal saturation indirectly implies no interference with amotosalen adsorption. Uncoupling the CAD from the original intent of pathogen reduction may allow the development of a “CAD‐only” component, salvaging otherwise non‐transfusable products with minimal additional cost. Future studies thoroughly studying the CAD to remove drugs may support revisiting donor deferral policies. Additionally, exploring the impact of the full INTERCEPT process with illumination would be necessary for pathogen‐reduced products.

Taken together, the CAD removed multiple drug classes and showed no saturation even at high drug concentrations, but the residual post‐CAD concentration may have a lower limit. This expanded study highlights a possible pathway to reevaluate drug deferrals based on when treated with a CAD. It may also safeguard transfusion recipients even when medications are not disclosed to a blood collector.

## FUNDING INFORMATION

This study was partially funded by the Cerus Corporation.

## CONFLICT OF INTEREST STATEMENT

The authors declare no conflicts of interest.

## Data Availability

The data that support the findings of this study are available from the corresponding author upon reasonable request.
